# Electrocardiographic diagnosis of acute myocardial infarction in a pacemaker patient: a case report

**DOI:** 10.1186/s12872-022-02462-7

**Published:** 2022-01-22

**Authors:** Xing Du, Yongjun Zhang

**Affiliations:** Department of Electrophysiology, Yijishan Hospital of Wannan Medical College, 2 Zheshan Road, Wuhu, China

**Keywords:** Acute myocardial infarction, Ventricular paced rhythm, Criteria, Cutoff, Non-concave ST-segment elevation

## Abstract

**Background:**

The electrocardiographic diagnosis of acute myocardial infarction (AMI) in the setting of cardiac pacing is often challenging. The original Sgarbossa criteria proposed in 1996 were demonstrated to be valid for diagnosis of AMI in both ventricular paced rhythm and left bundle branch block. To improve accuracy, the modified Sgarbossa criteria (MSC) were proposed.

**Case presentation:**

We presented a case of electrocardiographic diagnosis of AMI in a pacemaker patient. The Electrocardiogram (ECG) was false negative by using the original Sgarbossa criteria, whereas true positive by the MSC at a ratio of − 0.20.

**Conclusions:**

The application of MSC using an appropriate ratio (− 0.20 or − 0.25) may facilitate a timely diagnosis of AMI. Physicians should carefully choose the appropriate cutoff in a case-by-case basis.

## Background

The electrocardiographic diagnosis of acute myocardial infarction (AMI) during cardiac pacing is a long-standing confusion for clinicians. The original Sgarbossa criteria [1] proposed in 1996 were demonstrated to be valid for diagnosis of AMI in both ventricular paced rhythm and left bundle branch block. The criteria consist of ST-segment elevation equal to or greater than 1 mm concordant with QRS complex polarity in any lead (5 points); ST-segment depression equal to or greater than 1 mm in lead V1, V2 or V3 (3 points); ST-segment elevation equal to or more than 5 mm discordant with QRS complex polarity (2 points). Some evaluations of the original criteria have shown high specificity but poor sensitivity [2]. To improve accuracy, the modified Sgarbossa criteria (MSC) were proposed by Smith and co-workers [3]. Herein, we presented a case of electrocardiographic diagnosis of AMI in a pacemaker patient.

## Case presentation

**Fig. 1:**
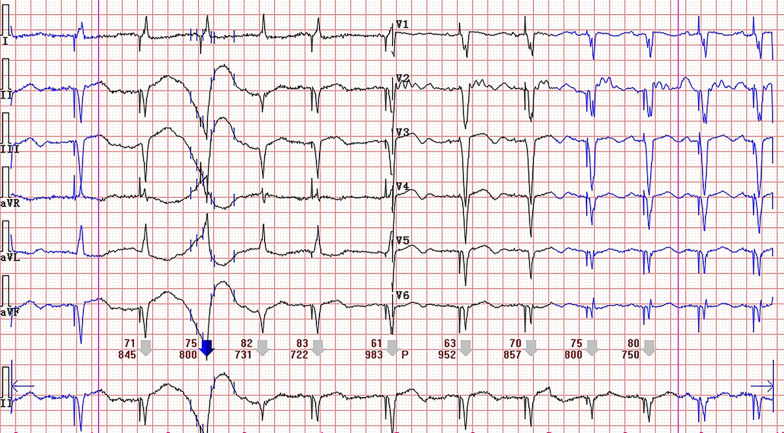
The baseline ECG showing a ventricular-paced rhythm with a heart rate of 74 beats/min

**Fig. 2:**
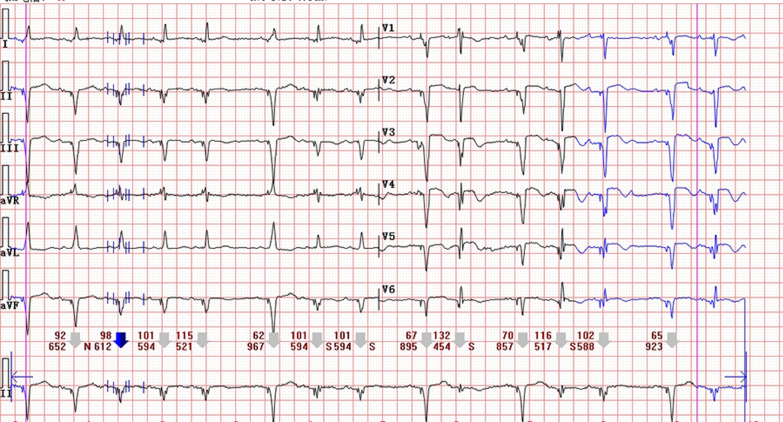
ECG showing sinus arrhythmia with ventricular pacing and atrial premature beat, discordant ST-segment elevation about 2 mm in leads V2, V3, V4 and less than 1 mm in lead V5, V6

**Fig. 3:**
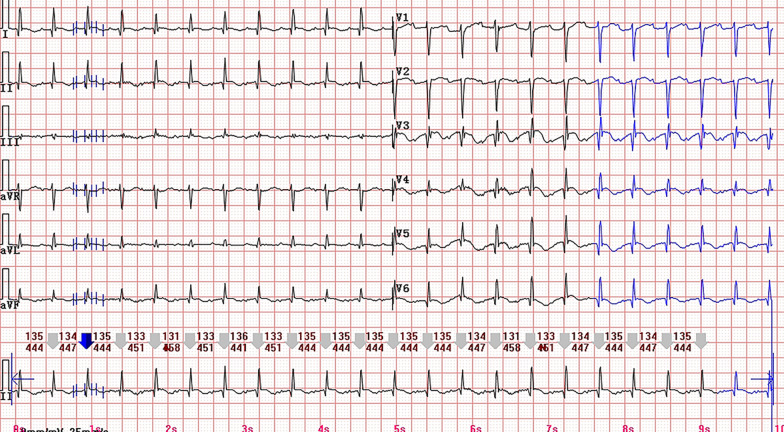
Sinus tachycardia with a heart rate of 134 beats/min, compatible with AMI

A 83-year-old woman was admitted to gastrointestinal surgery department with abdominal pain and distention for more than half a month. She had a medical history positive for pacemaker implantation and hypertension. The baseline Electrocardiogram (ECG) on admission showed sinus rhythm with ventricular pacing (Fig. 1). Based on the clinical symptoms and radiological findings of abdominal computed tomography (CT), she was diagnosed with ileus and underwent surgery. The fourth day after operation, the patient complained with chest tightness. A bedside ECG was recorded showing sinus arrhythmia with ventricular pacing and atrial premature beat. Ventricular fusion beats, spontaneous and full paced QRS complexes were presented on the ECG simultaneously. Discordant ST-segment elevation was about 2 mm in leads V2, V3, V4 and less than 1 mm in leads V5, V6 (Fig. 2). Although the new-onset horizontal ST-segment elevation didn’t meet the original Sgarbossa criteria, a suspected AMI couldn’t be ruled out. Subsequent laboratory test results showed cardiac troponin I, 2.81 ng/ml (reference range, 0–0.03 ng/ml), and creatine kinase isoenzyme MB, 40U/L (reference range, 0–25 U/L). A echocardiogram revealed regional wall motion abnormality and contractile dysfunction of left ventricular. The patient was subsequently treated with oral medicine (aspirin 300 mg, ticagrelor 180 mg, atorvastatin 40 mg) and hypodermic injection of enoxaparin 2000 AxaIU. A follow-up ECG showed sinus tachycardia at a heart rate of 134 beats/min, compatible with AMI (Fig. 3). Since sinus heart rate exceeded upper tracking rate, the pacemaker would not deliver a ventricular pacing stimulus to follow each P wave. Thus, spontaneous ventricular activation could show characteristic ECG manifestations of AMI. Finally, the patient underwent coronary angiography, demonstrated acute coronary occlusion of proximal left anterior descending artery, which was successfully implanted with one drug-eluting stent.

## Discussion and conclusions

Ventricular paced rhythm may obscure Q waves and cause secondary repolarization abnormalities on the ECG [4]. These changes can prevent accurate recognition of AMI in the setting of cardiac pacing, resulting in suboptimal outcomes [5]. Despite low sensitivity, the establishment of the original Sgarbossa criteria could be viewed as a significant advancement to handle this dilemma. Using of an absolute cutoff (5 mm) rather than a proportion criterion is one of the reasons for the low sensitivity [3]. Subsequently, in the MSC, an absolute criterion is replaced by a ratio of discordant ST-segment elevation to the preceding S-wave amplitude (ST/S ratio). The ST/S ratio less than − 0.25 supports a positive result [3]. According to the validation study by Meyers et al., the diagnostic characteristics of the MSC (with 80% sensitivity and 99% specificity) were superior than the original criteria (with 49% sensitivity and 100% specificity) [6]. Meanwhile, Meyers et al. had demonstrated that the MSC at a lower ratio of − 0.20 provided a 84% sensitivity and 94% specificity for diagnosing acute coronary occlusion. Hence, the MSC can facilitate diagnosis even if the original criteria are negative [7]. In our case, the maximum of discordant ST-segment elevation was about 2 mm. Obviously, this measurement did not meet the original Sgarbossa criteria. The values of ST/S ratio in precordial leads, especially in V2 and V3, were around − 0.22 to − 0.25 (depending on the beat-to-beat baseline variability), which suggested a negative result according to the MSC using a ratio of − 0.25. Conversely, the ECG would be true positive by using the cutoff of − 0.20, consistent with subsequent results of laboratory test, echocardiogram and coronary angiography.

It has been demonstrated that both the two cutoffs of the MSC performed well in the diagnosis of acute coronary occlusion. Using − 0.20 or − 0.25 as an appropriate cutoff usually depends on the clinical context and physician’s prediction. As for our case, the distinctive morphology of the ST-segment elevation might hold additional helpful information. Kosuge et al. [8] reported that ECGs from a majority of their anterior AMI patients were presented with non-concave ST-segment patterns. In patients with chest pain, a non-concave ST-segment elevation morphology manifested as convex or obliquely straight ST-segment elevation pattern strongly suggests AMI [9], while a concave morphology encounters in the non-infarction syndrome more frequently. Since both symptoms of chest pain and ECG with non-concave ST-segment elevation were encountered in our patient, using the MSC at a ratio of − 0.20 may be more superior to make the accurate diagnosis.

Immediate and accurate ECG identification of AMI in the setting of ventricular paced rhythym is critical to initiate appropriate reperfusion treatment. The application of MSC, which is more sensitive than the original criteria, may facilitate a timely diagnosis. Sometimes, using two different cutoffs (namely − 0.20 and − 0.25) of the MSC respectively might come to the exactly opposite conclusion. Physicians should carefully choose the appropriate cutoff in a case-by-case basis.

## Data Availability

All relevant data supporting the conclusions of this article are included within the article.

## Abbreviations

AMI: Acute myocardial infarction
MSC: Modified Sgarbossa criteria
ECG: Electrocardiogram
CT: Computed tomography
